# When the channel is the stressor: delivery medium in burnout interventions for healthcare students

**DOI:** 10.1080/10872981.2026.2745294

**Published:** 2026-10-07

**Authors:** Fitri Norhabiba, Chandra Kartika Dewi

**Affiliations:** a Department of Communication Science, Universitas Negeri Surabaya, Indonesia; b Biomedical Science, Faculty of Science and Technology, State UIN Sunan Kalijaga Yogyakarta, Indonesia

Dear Editor,

Burnout among healthcare students is now widely recognised, and the range of proposed remedies has grown accordingly. Biaigo et al. [1] offered a timely map of this field, charting ten intervention studies across medicine, nursing, and pharmacy, and rightly calling for longitudinal evidence. Their review showed that the field lacks an agreed hierarchy of interventions. Yet one pattern across the charted studies deserves closer attention: the medium through which an intervention reaches students may shape whether it works.

From a communication perspective, a delivery channel is never neutral. The smartphone is now both a common vehicle for well-being interventions and a documented source of cognitive strain. Experimental work shows that phone notifications alone significantly disrupted performance on an attention-demanding task, even when participants did not interact with the device [2]. A recent meta-analysis reports a link between problematic smartphone use and academic burnout, although findings across studies remain inconsistent [3]. An intervention delivered through the same device that interrupts study and sleep may therefore compete with the very stressor it aims to relieve.

The review's own data are consistent with this reading. In the mindfulness app trial, improvements in exhaustion and depersonalisation were no longer significantly different from control at week 10, with only personal accomplishment being sustained [1]. The app-delivered psychological flexibility trial found no significant differences in burnout between intervention and control groups [1]. By contrast, the intervention that combined a sunrise alarm clock with bedtime abstinence from electronic devices improved all three burnout dimensions [1], and its authors framed it around evidence that smartphone overuse is associated with higher exhaustion and depersonalisation among medical students [4]. These designs differ in duration, population, and rigour, so the contrast is suggestive rather than conclusive, but it is striking.

We propose calling the underlying variable channel congruence: the degree to which an intervention's delivery medium works with, rather than reproduces, the conditions it targets. The concept clarifies a tension in the review's discussion, which recommends that mobile applications for relaxation and meditation remain accessible long-term [1]. Long-term access through a device may be an advantage, but it may also extend exposure to notifications and the always-available communication that students already find draining. For a generation that studies, socialises, and rests through the same screen, where support arrives may matter as much as what it contains.

Mobile delivery has real strengths, including scale, low cost, and privacy, and Biaigo et al. are right to value accessibility. The stakes, however, are practical. If institutions adopt app-based wellbeing tools as an inexpensive default without examining channel effects, they may mistake the decay of short-term benefits for a limitation of the technique itself rather than of its medium. We therefore suggest that the longitudinal studies the authors call for code delivery modality as a potential moderator and that digital media use and notification exposure be added to the confounders the authors identify, alongside physical and mental health, socioeconomic status, family support, hobbies, and part-time employment. Comparing device-mediated and device-free versions of the same intervention would offer a direct test and help build the hierarchy of interventions the authors envision.

## Data Availability

Data sharing is not applicable to this article, as no new data were created or analysed in this study. All data discussed are available in the cited published sources.
